# Comparison of Traditional and Ultrasound-Guided Techniques for Vascular Access in Patients with Difficult Venous Access in Emergency Departments: Randomized Clinical Trial Protocol

**DOI:** 10.3390/nursrep15050177

**Published:** 2025-05-20

**Authors:** Mercedes S. Peralta-Gámez, Marina Gómez de Quero Córdoba, Silvia Reverté-Villarroya, Roser Cuesta-Martínez

**Affiliations:** 1 Emergency Department, Ávila Care Complex, Nuestra Señora de Sonsoles Hospital, Avenida Juan Carlos I, s/n, 05004 Ávila, Spain; mercedessegunda.peralta@estudiants.urv.cat; 2 Nursing Department, Seu Vilafranca, Universitat Rovira i Virgili, Carrera Amalia Soler, 29, 08720 Vilafranca del Penedés, Spain; 3 Research Group on Advanced Nursing (CARING)-161, Universitat Rovira I Virgili, 43002 Tarragona, Spain; mariaroser.cuesta@urv.cat; 4 Nursing Department, Campus Terres de l’Ebre, Universitat Rovira i Virgili, Avenue Remolins, 13–15, 43500 Tortosa, Spain; 5 Nursing Department, Campus Catalunya, Universitat Rovira i Virgili, Avinguda Catalunya, 35, 43002 Tarragona, Spain

**Keywords:** ultrasonography, peripheral venous catheter, difficult venous access, arterial blood gas analysis, emergency department, nursing care

## Abstract

**Background**: Vascular access in emergency departments (ED) is challenging for patients with difficult venous access (DIVA), causing delays and discomfort. Ultrasound-guided techniques may offer improved outcomes, making it crucial to assess their benefits, risks, and the effectiveness of validated identification systems. **Objectives**: To contribute new evidence regarding the effectiveness of validated tools for identifying DIVA and to assess the clinical benefits of ultrasound-guided vascular access in emergency care, and to assess their utility in arterial puncture for arterial blood gas sampling, from now on ABG, within the ED. **Methods**: This study follows the Standard Protocol Items: Recommendations for Interventional Trials (SPIRIT) guidelines for protocol development and the Consolidated Standards of Reporting Trials (CONSORT) guidelines for the conduct and reporting of the randomized clinical trial (RCT). The trial will be conducted in Spain throughout 2025. The study population will consist of 114 subjects with difficult intravenous access (DIVA), identified using the DIVA scale for individuals under 14 years of age and the A-DICAVE scale for adults, along with 80 subjects from the general surgical area (GSA). Participants will be randomly assigned, in a 1:1 ratio, to either the Control Group (CG) (traditional technique) or the Experimental Group (EG) (ultrasound-guided technique). Data collected will include sociodemographic characteristics, procedure-related variables (e.g., time required, human resources, and materials used), as well as pain levels, assessed using validated scales (EVA, FLACC, PAINAD), and overall satisfaction from both patients and healthcare professionals. Ethical approval has been obtained, and the trial will be registered as an RCT through an official clinical trial registry before recruitment begins. **Results**: Expected results suggest ultrasound guidance will significantly improve first-attempt success rates, reduce procedural time, enhance patient comfort, and optimize resource utilization compared to traditional techniques. **Conclusions**: The integration of ultrasound-guided vascular access into routine emergency protocols could enhance patient safety, satisfaction, and procedural efficiency in emergency care settings.

## 1. Introduction

Approximately 90% of patients who attend emergency departments (EDs) require venipuncture, with short peripheral intravenous catheters (PIVCs) being the most commonly used invasive devices in these settings [1]. Despite its routine nature, vascular access can be particularly challenging in a subset of patients, even for experienced healthcare professionals. When two or more attempts are required to achieve successful cannulation, patients are typically classified as having difficult intravenous access (DIVA) [2]. Repeated failed attempts are associated with damage to venous and arterial structures, increased pain and distress for the patient, delays in diagnosis and treatment, and greater consumption of healthcare resources [3,4]. Several studies have examined the clinical and demographic factors associated with DIVA. Conditions such as obesity, hypotension, generalized edema, advanced age, and the presence of multiple hematomas have been frequently cited as risk factors for cannulation failure [5,6]. Among adult populations, the prevalence of DIVA has been estimated to range between 10% and 24% [1]. In pediatric settings, the prevalence is even higher, with reports indicating that approximately 37% of children may fall into this category [7].

To address the challenges of early identification, a number of clinical tools and scoring systems have been developed. The first of these, the DIVA scale, was validated by Yen et al. for use in pediatric populations and aimed to guide clinical decision making from the point of triage [8]. This approach was later expanded by Kuensting et al., who incorporated additional variables such as the patient’s clinical history, the anticipated treatment plan, and the skill level of the staff performing the procedure [9].

In adult populations, the A-DIVA scale, introduced by van Loon et al. in 2016, provided a framework for identifying patients at risk of difficult venous access in surgical settings [10]. More recently, Salleras et al. developed the A-DICAVE scale, a simplified and ED-appropriate tool designed to support early recognition of DIVA and promote the use of ultrasound-guided techniques in these cases [11,12].

Despite increased attention to peripheral venous cannulation, few studies have investigated the early identification of patients with anticipated difficulties in arterial puncture, such as those requiring arterial blood gas (ABG) sampling. In these cases, ultrasound-guided techniques offer an opportunity to improve procedural success, enabling better visualization of target vessels and real-time confirmation of needle and catheter placement [13,14].

Although the benefits of ultrasound guidance in vascular access are well supported by evidence, its implementation in routine ED practice remains limited. Most studies have focused on critical care or surgical patients, or on central line placement, rather than peripheral access in general ED populations [13,15,16,17]. Moreover, the absence of standardized protocols and variability in staff training continues to be barriers to broader adoption [18,19].

In Spain, vascular access is considered a core nursing competency taught during undergraduate education, as stipulated in Law 16/2003 on the cohesion and quality of the National Health System. However, there is no unified national standard for nurse training in ultrasound-guided vascular access. Some institutions have developed internal training programs that have demonstrated favorable outcomes, showing that nurses can effectively perform ultrasound-guided PIVC insertion following specific education [20,21,22]. 

Several authors have argued that this training should begin during undergraduate education to better prepare future professionals [23].

This randomized clinical trial protocol aims to contribute new evidence regarding the effectiveness of validated tools for identifying DIVA and to assess the clinical benefits of ultrasound-guided vascular access in emergency care. In addition to evaluating procedural success, the study will assess pain associated with the technique and satisfaction levels reported by patients, families, and healthcare providers.

### Hypothesis and Objetive

The ultrasound-guided puncture facilitates vascular access in patients with difficult vascular conditions, identified using validated scales, who attend emergency departments.

The main objective of this study is to create a protocol for an RCT to compare ultrasound-guided techniques with traditional methods of vascular access in patients with DIVA identified by validated ED scales.

## 2. Materials and Methods

The study is designed as an open-label, randomized controlled trial (RCT) to be conducted in the emergency department (ED) of a secondary-level hospital in Spain. Recruitment is expected to begin in January 2025, with data collection concluding in December 2025. The trial protocol has been developed in accordance with the Standard Protocol Items: Recommendations for Interventional Trials (SPIRIT) [24] guidelines, ensuring a comprehensive and transparent study design. Upon completion, the results will be reported following the Consolidated Standards of Reporting Trials (CONSORT) [25] guidelines to ensure clarity and reproducibility. This protocol will be registered with ClinicalTrials.gov prior to participant enrollment.

### 2.1. Scope of the Study

The study will be carried out in the ED of the Hospital Nuestra Señora de Sonsoles (HNSS) de Ávila, belonging to the Regional Health Management of Castilla y León, Spain. This center has a catchment area of 158,140 inhabitants [26], which are spread over a total of 469 localities [27].

This population has an average age of 47.99 years with a dependency ratio of 42.56% for those under 65 and 63.20% for the total population. The population over 65 years of age constitutes 26.08% of the total population [26].

The HNSS ED receives about 47,000 emergencies per year (about 5000 pediatric patients), a figure that is increasing year after year. Some 34 nurses work in the ED in morning/evening/night shifts. The number of nurses per day is 6/7/5, respectively [28]. The nurses in the service have the official nationally recognized qualification, and therefore the necessary competences to perform vascular punctures. Prior to the study proposal, 99% were trained in the use of ultrasound-guided puncture by in-house training provided by nurses from the department who had acquired their skills and experience externally through regulated training.

### 2.2. Design

**Figure 1:**
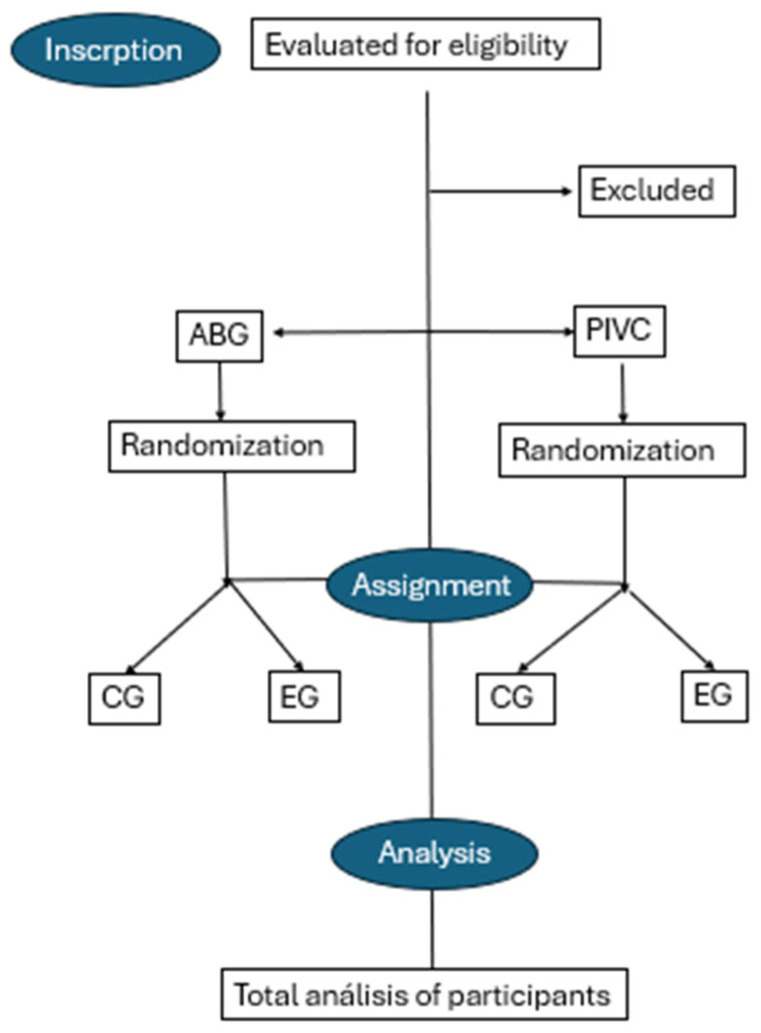
Flow chart RTC. Produced by the authors.

The RCT will collect data derived from the procedures performed on patients selected through probabilistic random sampling. The study workflow is outlined in the following flowchart (Figure 1). Following the CONSORT criteria, after an initial identification of the possible participants in the study, the inclusion and exclusion criteria will be applied, in order to subsequently assign each subject to the relevant group depending on the technique to be performed (PIVC or arterial punctures). Once the technique has been selected, using a randomized system, the subjects will be assigned to the Control or Experimental Groups, and the technique will be performed using the relevant procedure, using the traditional technique for CG and an ultrasound-guided technique for SG. After obtaining the required number of samples, the data obtained shall be analyzed.

### 2.3. Participants

The study will recruit DIVA patients identified using the A-DICAVE scale for patients aged > 14 years and the DIVA scale for those aged < 14 years, who attend the ED and require short PIVC or AGB.

Inclusion criteria:

- Prescription for CIP or GA cannulation.
- Informed consent signed by the patient or patient’s representative.
- A score ≥ 3 on the A-DICAVE scale or ≥4 on the DIVA scale for PIVC.

Exclusion criteria:

- Scores below 3 on the A-DICAVE scale or below 4 on the DIVA scale for PIVC.
- Inability to obtain informed consent from the patient or patient’s representative.

### 2.4. Sample Size

The sample size calculation will be based on the minimum sample size formula for mean comparison. Considering data from a similar study conducted in other healthcare settings [6] and assuming a minimal detectable mean difference of 0.52 and a variance of 1.64 (“number of attempts” variable), a minimum sample size of 95 participants was obtained. Accounting for a 20% loss rate, the required sample size increases to 114 participants, with 57 in each group.

For the sample size required for arterial puncture, the calculation will follow data from a study by Grandpiere et al. [3], which reported a 34% difference in success rates using ultrasound compared to the traditional technique. To demonstrate a minimum difference of 37% in the first-attempt success rates between the two groups, with an alpha error of 0.5%, 90% power, and accounting for a 20% loss rate, the required sample size is 80 participants, with 40 in each group.

### 2.5. Randomization of the Sample

Random assignment of participants to the Control Group (CG) and the Experimental Group (EG) will be conducted with a 1:1 ratio using a simple randomization method. Each participant will have an equal probability of being assigned to either group, minimizing bias and ensuring the equitable distribution of characteristics between the groups. To achieve this, a computer-generated random number sequence will be used. Additionally, random permuted blocks will be created to reduce the predictability of the random sequence, while ensuring an equal number of subjects in each group.

Those responsible for carrying out the techniques shall be unaware of the randomization code so that their performance is not constrained.

Details of the sample selection, inclusion criteria applied, and methods used for random assignments to CG and EG will be documented. This will ensure participants’ right to withdraw their data and guarantee the transparency and replicability of the study.

### 2.6. Statistical Methods

The data will be analyzed using SPSS software, version 22. Initially, normality tests will be performed to assess the distribution, homogeneity, and comparability of the sample, employing kurtosis, skewness coefficients, and the Shapiro–Wilk test. A descriptive analysis will then be conducted using measures of central tendency and dispersion (SD) for quantitative variables, alongside calculations of the median and interquartile range (IQR). Qualitative variables will be expressed as frequencies (n) and percentages (%).

Subsequently, appropriate statistical tests will be conducted to determine whether the hypothesis can be rejected, analyzing relationships between the different variables. For qualitative variables, the Chi-square test will be used, or Fisher’s exact test if values < 5 are observed in the sample. For quantitative variables, Student’s t-test will be applied, or the Mann–Whitney U test in cases where the data do not follow a normal distribution.

In all calculations performed in the study, an alpha error of 0.05 and a beta error of 0.2 will be considered, with a 95% confidence level.

### 2.7. Ethical Considerations

The study has received approval from the Ethics Committee for Research and Medicines (GASAV/2024/60) and consent from the local healthcare management authorities where the study will be conducted. The research will adhere to the ethical principles outlined in the Declaration of Helsinki and the World Medical Association’s six ethical principles for medical research involving human subjects.

The study will comply with the General Data Protection Regulation and Spain’s Organic Law 3/2018 on Data Protection, ensuring the confidentiality and anonymity of all participants. The provisions of Spanish Law 41/2002 on patient autonomy will also be respected, particularly in cases involving children and patients with cognitive impairments.

Participants will be provided with all necessary information about the study via a Participant Information Sheet and a copy of the informed consent form, which will include the contact details of the principal investigator.

## 3. Expected Results

Data from the CG will be obtained using the traditional technique, which involves palpation and visualization of veins following the application of a tourniquet to the selected limb for PIVC and pulse palpation for arterial punctures. For the EG, the ultrasound-guided technique will be employed, beginning with vessel mapping using the rapid peripheral vein assessment (RaPeVa) technique, the identification and measurement of the selected vessel, and an examination of surrounding structures to avoid potential injury. The ultrasound machine available for performing the ultrasound-guided technique will be a SonoSite model M-turbo, together with a 13-6MHz linear transducer. If the practitioner deems it necessary, they may switch techniques, recording the change on the data collection form and accounting for both interventions.

Table 1 shows the steps to be followed for ultrasound-guided punctures in the EG. This table follows European recommendations on the correct indication and use of peripheral venous devices of the WoCoVa [16] and the Osakidetza arterial puncture procedure guide for blood gases in the adult population [29].

**Figure 2:**
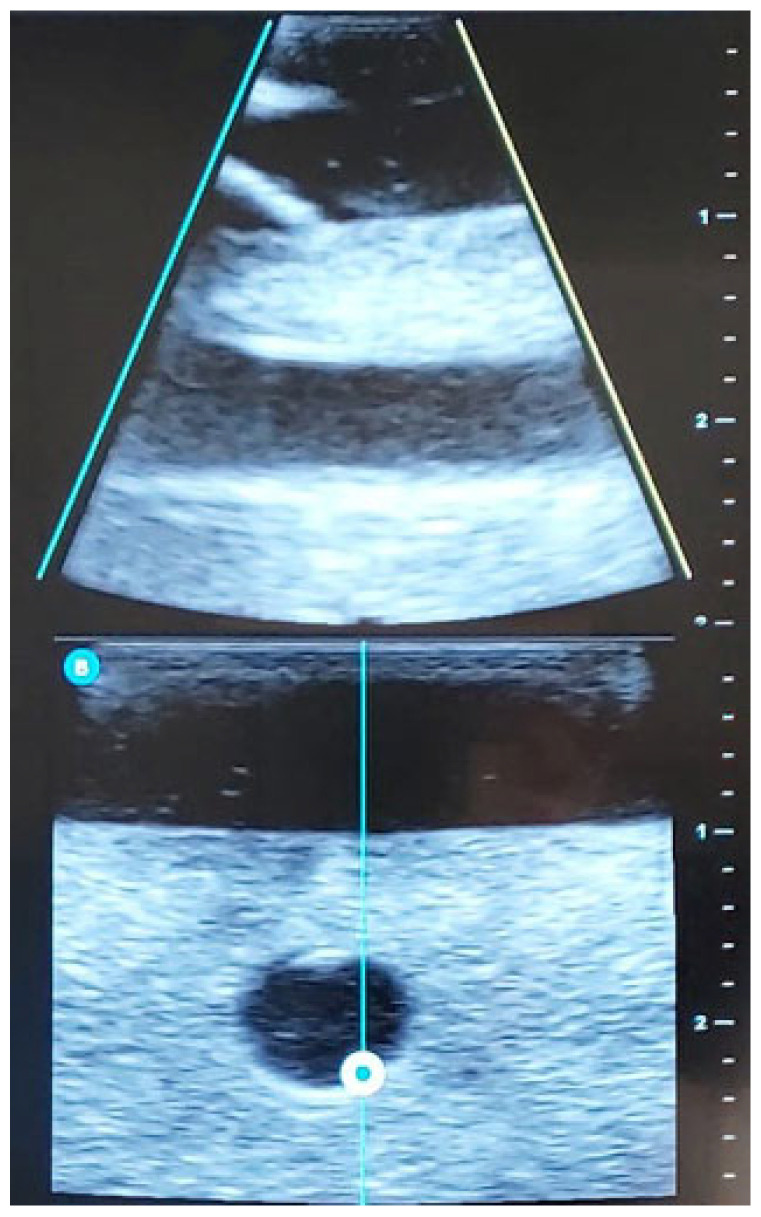
Ultrasound image of venous access. *Longitudinal plane. (Own source)*.

**Figure 3:**
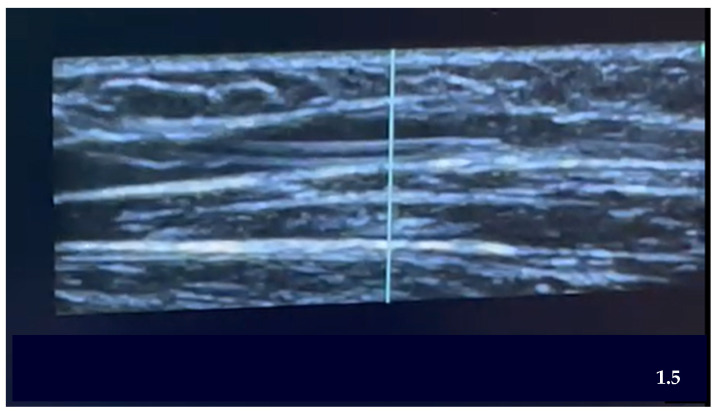
Ultrasound canalization PIVC. *Transversal Plane. (Own source)*.

Below are images of the visualization of the vessels using ultrasound (Figure 2) and the placement of the PIVC within the selected vein (Figure 3).

The study design does not allow for the blinding of the technique assignment, either for participants or the collaborating nursing professionals. However, an external observer will be responsible for collecting data regarding patient and/or family pain and satisfaction. All data collected will be alphanumerically coded to ensure that analysts remain blinded to the treatment group assignments.

### 3.1. Measurement Instruments

To identify DIVA patients during PIVC catheterization, two scales will be used depending on the patient’s age:

For patients aged ≥14 years, the A-DICAVE scale [11] will be applied. This scale consists of three items: vein visualization (scored 0–2), vein palpation (scored 0–2), and a history of puncture difficulty (scored 0–1). The total score is categorized as follows: easy (0–2 points) and difficult (3–5 points).

For patients aged <14 years, the DIVA4 scale [2] will be used. This scale, scored 0–10, includes four items: visible vein after tourniquet application (0–2 points), palpable vein after tourniquet application (0–2 points), age (≥3 years: 0 points; 1–2 years: 1 point; <1 year: 3 points), and history of prematurity with gestational age < 38 weeks (0–3 points). It has been demonstrated that scores > 4 typically indicate the need for multiple punctures to achieve adequate venous access.

In order to assess the level of satisfaction of professionals, patients and their families, an ad hoc instrument will be used, based on a 5-point Likert scale, ranging from 1 = “not at all satisfied” to 5 = “very satisfied”. Each item will be rated with a value from 1 to 5. To determine the general level of satisfaction, the following ranges of interpretation have been established:

1. Not at all satisfied.
2. Not very satisfied.
3. Satisfied.
4. Quite satisfied.
5. Very satisfied.

To assess the satisfaction levels of professionals, patients, and their families, a 5-point Likert satisfaction scale will be applied. If patients are unable to express their satisfaction, only the response from their family members will be recorded.

Pain assessment will utilize numerical scales (0–10):

○ For adults and children aged > 7 years, the Visual Analogue Scale (VAS) [30] will be used.
○ For children aged < 5 years, the FLACC scale (Face, Legs, Activity, Cry, Consolability) [31] will be applied.
○ For adults with cognitive impairments, the PAINAD scale (Pain Assessment in Advanced Dementia) [32] will be employed.

Once the corresponding technique has been carried out, the data from the measuring instruments will be recorded through the form that will have the necessary items for their evaluation, together with the rest of the variables, as shown in Table 2.

### 3.2. Data Collection System

The collaborators will be provided with the necessary training for data collecting. To ensure the quality of care and patient safety with respect to the techniques to be developed in the study, the collaborators must be nurses from the service who have received specific training in ultrasound-guided puncture and have a minimum experience in the service of 2 years, thus being considered competent nurses according to Patricia Benner’s model [33].

Collaborators will be provided with a data collection form accessible from the point of care. A separate form will be used for each technique (PIVC or ABG), ensuring that the data are transferred to a dedicated database specifically prepared for the study. The database will record all relevant variables (Table 2).

The study will be coordinated by the principal investigator, attached to the ED of the hospital, who will be responsible for compliance with the protocol, data integrity, and the supervision of the research team.

The governance of the project will be overseen by an Internal Control Committee, composed of members of the emergency department, which will meet quarterly to review study progress, ethical compliance, and adverse events.

The involvement of an external monitoring committee is not foreseen, given the low-risk nature of the study.

All adverse events shall be documented in a specific data collection form (CRF), with details on their nature, severity, duration, treatment required and outcome.

Adverse events will be considered any unanticipated clinical incident temporally related to the venous or arterial puncture procedure, such as: extensive hematoma, local infection, phlebitis, persistent pain, or vascular damage. These events shall be recorded by the investigator immediately after the procedure and during the clinical follow-up of the patient in the emergency department. An intermediate safety analysis will be carried out when 50% of the planned recruitment is reached.

## 4. Discussion

An extensive review of the literature was conducted to ensure this study protocol addresses current gaps in evidence, offering new insights into DIVA patient identification and the use of ultrasound-guided vascular access techniques in emergency departments (ED). This protocol integrates validated identification scales for DIVA, enhancing the reliability of patient categorization and improving clinical outcomes.

In ED settings, few studies have employed validated methods for identifying DIVA patients in conjunction with ultrasound-guided techniques from the outset. Most existing studies rely on subjective observations, such as palpable veins or the number of attempts required for successful venipuncture. Notable works, including those by Rodríguez-Herrera et al. [19] and Rubiera [34], have highlighted the potential of early identification in improving procedural success rates. The A-DICAVE scale, validated by Dr. Salleras in 2020 [15,35], is among the few scales developed specifically for this purpose, yet its adoption in practice has been limited. To address this, our protocol incorporates the A-DICAVE scale to provide a standardized, objective approach for identifying DIVA patients.

Research comparing traditional and ultrasound-guided peripheral intravenous catheter (PIVC) insertion in EDs has consistently shown that ultrasound guidance improves first-attempt success and reduces the number of attempts required. Studies by Rubiera et al. [34] and Rodríguez-Herrera et al. [19] support these findings, yet these studies often lacked probabilistic sampling or failed to control for important variables like the use of medications through PIVCs. In contrast, our study accounts for such variables and includes confounding factors such as the cognitive state of patients, which may influence procedure outcomes.

Although patient satisfaction with ultrasound-guided techniques has been assessed in various studies [36], practitioner satisfaction remains underexplored. Given its impact on the adoption of new techniques in clinical practice, our protocol incorporates the evaluation of practitioner satisfaction as an additional key variable.

Evidence supporting the benefits of ultrasound-guided PIVC insertion is robust, with a meta-analysis by Tran et al. [37] demonstrating a significant improvement in first-attempt success rates. However, these studies were not conducted in ED settings, highlighting the need for research tailored to the unique demands of EDs. Our protocol aims to fill this gap by focusing on ED populations, where challenges such as high patient turnover and time constraints are prevalent.

Regarding pain assessment, while tools like the Visual Analog Scale (VAS) have been used to measure discomfort, they typically exclude pediatric patients or those with cognitive impairments. To ensure a comprehensive evaluation, our study includes alternative pain assessment scales suitable for a broader patient population, including those with cognitive challenges.

The use of ultrasound for arterial punctures in EDs remains underexplored, with most research focused on critical care or anesthesia settings [38,39]. However, studies such as that by Grandpiere et al. [3] have demonstrated that ultrasound guidance reduces the number of attempts and associated pain in difficult arterial access cases. Our study builds on this evidence, extending its focus to include patients with cognitive impairments and healthcare professional satisfaction.

The benefits of ultrasound-guided techniques extend beyond clinical outcomes, leading to a reduction in resource usage. Fewer attempts reduce material consumption and optimize staff allocation, contributing to faster procedures, shorter ED stays, and quicker test results [6,11,40,41,42]. Additionally, the increased efficiency enhances healthcare professional satisfaction and improves overall workflow in EDs.

While this study may face limitations, such as the inability to implement a double-blind design, random sampling and data coding ensure the reliability of our findings. The single-center design could also limit generalizability; however, the protocol’s methodological rigor allows for adaptation to other centers, providing flexibility for future studies.

In summary, this study addresses critical gaps in the literature by incorporating validated DIVA identification scales and ultrasound-guided techniques for PIVC and arterial access in ED settings. By considering confounding variables and focusing on a representative patient sample, our research aims to provide valuable evidence to enhance ED practices and improve patient care.

## 5. Conclusions

This study protocol will allow for the development of a study to evaluate the effectiveness of ultrasound-guided vascular access compared to traditional methods in DIVA patients identified by validated scales in the ED. After the implementation of this protocol, it is expected that ultrasound guidance will optimize ED workflows, improve patient comfort, and reduce resource consumption. If proven effective, ultrasound-guided techniques should be incorporated into standard ED protocols to improve safety, patient satisfaction, and clinical efficiency.

## Figures and Tables

**Table 1:** Steps of the EG ultrasound-guided technique.

Steps of the EG Ultrasound-Guided Technique	
**STEP 1**	Patient identification and procedure explanation.
**STEP 2**	Preparation of necessary materials.
**STEP 3**	Ultrasound-guided examination of the limb to be punctured (RaPeVa): Identification of vessel patency. Identification of nearby structures to prevent injury during puncture.
**STEP 4**	Selection of an appropriate vessel (depth, diameter, and patency): PIVC: Vessel at a depth of <5 mm, with a diameter at least two-thirds larger than the catheter. Avoid areas of joints or bony prominences. ABG: Depth appropriate for the needle used.
**STEP 5**	Preparation of the puncture site: Hand hygiene. Skin disinfection with 2% alcoholic chlorhexidine (2% aqueous chlorhexidine for patients under 2 years old). Non-touch technique.
**STEP 6**	Ultrasound-guided puncture: Appropriate angle based on vessel depth and puncture system. PIVC: Verification of catheter tip placement using ultrasound imaging. ABG: Visualization of the needle inside the artery.
**STEP 7**	PIVC: Verification of catheter patency, placement of an extension system, and catheter fixation with a transparent, semi-permeable dressing. ABG: Arterial blood sample collection, followed by application of pressure at the puncture site for 5 min (10 min for anticoagulated patients).

**Table 2:** RTC variables. Source: produced by the authors.

RTC Variables	
**Dependent Variables**	**Description**
Type of technique	Arterial puncture, peripheral venous cannulation with short PIVC
Total score on the A-DICAVE scale	From 0 to 10 score in patients below 14
Score in DIVA scale	From 0 to 8 score in patients over 14
Score of every item on the A-DICAVE scale	Score of every item on the scale
Success	Successful result (Yes/No)
Number of attempts	Number of punctures attempts to achieve cannulation
Numbers of nurses attempting	Number of nurses to achieve cannulation
Time taken in the technique	Time in minutes taken for the procedure
Cannulation zone	Pace of insertion of the catheter (hand, forearm, flexure, arm)
Catheter gauge	Measurement in gauges of the used catheter
Pain	EAV scales (oriented patient), FLACC (pediatric patient) or PAINAD (disorientated patient)
Permanence of the vascular access	Permanence time of the catheter
Secondary effects	Type of effects (extravasation, phlebitis, obstruction, others)
Patient satisfaction	Level of satisfaction (5-point Likert scale)
Family satisfaction	Level of satisfaction of the family (5-point Likert scale)
Professional satisfaction	Level of satisfaction of the professional who applied the technique (5-point Likert scale)
**Independent variables**	**Description**
Age	Years
Gender	Male, female, nonbinary
Reason for the consultation	Reason for the assistance according to nursing record
Emergency zone	Assigned emergency zone
Triage level	Classification SET (I, II, III, IV, V)
Predisposing factors of DIVA	Factors such as addiction, obesity, old age among others
**Procedure variables**	**Description**
Type of technique	Traditional, ultrasound guided
Transducer placement	Longitudinal, transversal, none
**Confusion variables**	**Description**
Medication given	Medication used through the access
Dilution volume	Volume in ml of the medication dilution
Time of administration	Taken time in minutes of the administration
Conscience level	Glasgow scale
Agitation	Yes/No

## Data Availability

Data sharing is not applicable to this article.

## Public Involvement Statement

No public involvement in any aspect of this research.

## Guidelines and Standards Statement

This manuscript was drafted against the CONSORT (a checklist of information to include when reporting a randomized trial).

## Abbreviations

The following abbreviations are used in this manuscript:

AGB	Arterial puncture for Artery Blood Gas sampling
CG	Control Group
DIVA	Difficult venous access
ED	Emergency Departments
EG	Experimental Group
FLACC	Face, Legs, Activity, Cry, Consolability
HNSS	Hospital Nuestra Señora de Sonsoles
IQR	Interquartile range
n	Frequencies
PAINAD	Pain assessment in advanced dementia
PIVC	Peripheral intravenous catheter
RaPeVa	Rapid peripheral vein assessment
RCT	Randomized Clinical Trial
SD	Dispersions
VAS	Visual analogy scale
